# Jealousy, sexism, and romantic love myths: the role of beliefs in online dating violence

**DOI:** 10.3389/fpsyg.2023.1212737

**Published:** 2023-09-04

**Authors:** Daniela Ramírez-Carrasco, Rodrigo Ferrer-Urbina, Felipe Ponce-Correa

**Affiliations:** ^1^Escuela de Psicología y Filosofía, Universidad de Tarapacá, Arica, Chile; ^2^Facultad de Medicina, Universidad de Tarapacá, Arica, Chile

**Keywords:** dating violence, cyber-violence, cyber-harassment perpetrated, cyber-victimization, jealousy, sexism, romantic love

## Abstract

With the massification of the Internet and social networks, a new form of dating violence called cyber-violence has emerged, which involves behaviors of control, humiliation, intimidation and threats towards the partner or ex-partner. Using a non-probabilistic sample of 1,001 participants aged 18 to 25 years, the present study used an *ex post facto*, retrospective, cross-sectional, single-group design to analyze the joint effects that beliefs associated with dating violence such as romantic love myths, jealousy, and sexism have on the victimization and perpetration of cyber-violence. The results evidenced that jealousy is involved in both Cyber-victimization and Cyber-harassment perpetrated, while sexist beliefs are only involved in perpetration. In the discussion section, it is postulated that cyber-violence is a phenomenon that is more related to the probability of aggression, but not to the probability of being a victim. Finally, limitations and implications for future research are discussed.

## 1. Introduction

Dating violence (DV), as it is referred to in Anglo-Saxon literature, refers to any aggressive behavior, harassment, or intentional attack, towards a partner or ex-partner, either physically, sexually, or psychologically, occurring in a romantic relationship involving young people or adolescents [Centers for Disease Control and Prevention (CDC), 2020], who do not have, nor have had, legal ties, economic dependence, or situations of mutual cohabitation (Vizcarra et al., 2013; Pazos Gómez et al., 2014; Rubio-Garay et al., 2017).

Early studies focusing interest on violence in these age groups date back to the mid-1980s (e.g., Makepeace, 1981; O’Keeffe et al., 1986), although they have become more relevant in the last two decades (Rodríguez-Díaz et al., 2017), being recognized as a severe public health problem (Valdivia Peralta and González Bravo, 2014), considering its notorious impacts on the physical and mental health of young people (Exner-Cortens et al., 2013), and its high prevalence (López-Barranco et al., 2022).

The emergence and massification of the Internet and social networks has revealed that dating violence can be exercised both in person and through digital media [Centers for Disease Control and Prevention (CDC), 2020], resulting in a new form of dating violence, called cyber-violence in relationships or online dating violence, which refers to violent behaviors towards the partner or ex-partner through the use of new information and communication technologies (ICTs), such as the Internet, cell phones and social networks (Guadix et al., 2018) and is characterized by including behaviors of control, humiliation, intimidation, and threat (Cava et al., 2020a,b), such as, for example, checking the cell phone, sharing photos, videos, and information of the partner without consent, threatening exposure of content on social networks, limiting the content that their partner uploads to the Internet, exerting control over the partner to monitor their social relationships, knowing where they are and what they are doing, among others (Rodríguez et al., 2021). These behaviors impact the psychosocial well-being of young people (Cava and Buelga, 2018; Cava et al., 2020a,b), leading several adverse consequences in the short and long term (Pérez-Marco et al., 2020; Tomaszewska and Schuster, 2021).

Young people and adolescents may be involved as victims of cyber-violence in their romantic relationships, i.e., being subjected to virtual control or abuse by their partner or ex-partner or perpetrating such violent behaviors (Marcos et al., 2020). However, it is typical for this type of situation to occur with a bidirectional character, where they can be, at the same time, victims or aggressors (López-Barranco et al., 2022). Therefore, when evaluating cyber-violence, it is relevant to analyze it from both roles, which may allow identifying common or differential factors in the levels of cyber-violence exercised and received.

Numerous studies have been developed in recent decades focused on identifying cyber-violence behaviors (e.g., Zweig et al., 2013), their prevalence (Peskin et al., 2017; Smith et al., 2018), and the consequences of such actions (Shorey et al., 2012; Borrajo and Gamez-Guadix, 2016; Hancock et al., 2017; Lu et al., 2018). However, given the grueling consequences for the physical and mental health of young people and adolescents, in recent years, and with a focus on prevention, interest has centered on identifying those factors that increase or decrease the likelihood of being a victim or perpetrator of cyber-violence (Vizoso-Gómez and Fernández-Gutiérrez, 2022).

Many young couples and adolescents are unable to recognize violent behaviors and confuse them as demonstrations of romantic love (Pérez-Marco et al., 2020), normalizing the use of cyber-violence as a strategy to resolve their conflicts (Vizoso-Gómez and Fernández-Gutiérrez, 2022). Conflict is a constitutive aspect of any relationship, but it can trigger diverse and intense negative emotions (Chapman and Gillespie, 2019). Negative emotions have been identified as a central precedent of a violent response, evidenced as the main reason young people and adolescents explain aggression in their relationships (Pazos Gómez et al., 2014). Conflicts can have diverse origins; however, given that in youth and adolescent relationships, economic or domestic commitments are not frequently present (Garzón González et al., 2017), the primary source of conflict is associated with jealousy (Alegría del Ángel and Rodríguez Barraza, 2015), which, at the same time, are also considered as a direct motivation to exercise dating violence (Rodríguez-Domínguez et al., 2018).

Jealousy occurs in response to the perception of threat, real or imagined, of loss or damage of a significant relationship (Branson and March, 2021), as a consequence of the involvement of a third person, which translates into a negative emotional response marked by anger, sadness, and fear, among others (Bosch et al., 2008). Although jealousy is commonly identified as a pathological response that should be avoided, multiple cultural beliefs act as a justification and reinforcer of jealousy, being typical for it to be normalized, supported by beliefs such as the myths of romantic love (Yela, 2000), and gender role (i. e. sexism; Rodríguez-Domínguez et al., 2018), approaches that could contribute to young people normalizing certain types of behaviors and even perceiving them as an expression of love (Malonda et al., 2017; Garrido and Barceló, 2019).

Romantic myths are a set of commonly shared socially beliefs about the how “real love” and meaningful romantic relations must be, in words of Yela (2003), “the supposed true nature of love.” As happens with other myths, they are usually fictitious, absurd, misleading, irrational, and impossible to fulfill (Yela, 2003; Bosch et al., 2008). Myths about romantic love have been recognized as a factor that contributes to fostering and maintaining violence in relationships (Garrido, 2001; González Méndez and Santana Hernández, 2001; Sanmartín et al., 2003; Lagarde, 2005; Sanpedro, 2005; Bosch et al., 2008; Bonilla-Algovia and Rivas-Rivero, 2018; Del Castillo, 2018; Bonilla-Algovia and Rivas-Rivero, 2021; Ariza Ruiz et al., 2022), as they reinforce violent love models by promoting the idea that true love should be possessive and exclusive (Yela, 2003; Pequeño et al., 2019).

Sexism is a multidimensional construct, referring to sexist beliefs and attitudes that limit gender roles and expression by sex, usually associated with discrimination against women (Glick and Fiske, 1996; Fernández-Montalvo and Echeburúa, 1997; Arnoso et al., 2017). During the last decades, sexist beliefs have been considered a relevant vulnerability factor for the perpetration and victimization of violence (León-Ramírez and Ferrando Piera, 2014; Ibabe et al., 2016; Marcos et al., 2020), being considered as one of the main background in the justification and promotion of dating violence (Orozco Vargas et al., 2022), as it that sexism can contribute to power inequality in the relationship and to the reinforcement of stereotypical gender roles (Glick and Fiske, 2001), which are also present in cyber-violence, where online harassment and abuse can be motivated by misogyny and sexism. Some research in the young and adolescent population indicates that men, and women with more traditional beliefs of sexism, are more accepting of the use of aggression in couple relationships and aggression towards women (Ulloa et al., 2004), data that confirm the existence of sociocultural factors that influence and reinforce sexist models and gender differences (Soler et al., 2005; Pazos Gómez et al., 2014).

In summary, jealousy, romantic love myths, and sexism are interrelated components that would constitute a belief system that would favor the emergence of negative emotions and legitimize violent manifestations (Cava and Buelga, 2018), including those that are expressed virtually, even though the evidence confirming these relationships is robust, most available studies present restrictions to understanding the joint role of these variables and establishing their relative importance since they are restricted to a few variables (e.g., De Los Reyes et al., 2022). In addition to the methodological restrictions of the use of univariate models and the lack of multivariate approximations, and the one that is of most significant interest for the present study, most of the available studies have focused on manifestations of face-to-face violence, which limits the generalizability of the findings to the new dynamics of romantic relationships, which have a robust virtual component.

In this scenario, it seems relevant to have evidence that establishes the combined effects of various beliefs associated with dating violence on cyber-violence in relationships to contribute to developing and prioritizing preventive actions from psychology and promoting healthy relationships. Therefore, the present study contrasts an explanatory model of online dating violence for cyber victimization and Cyber-harassment perpetrated from a latent variable model that includes romantic love myths, sexism, and jealousy.

## 2. Materials and methods

### 2.1. Study design

This research corresponds to a non-experimental, *ex post facto*, retrospective, cross-sectional, single-group, correlational study with non-probabilistic sampling via social networks.

### 2.2. Participants

The initial sample consisted of 1,360 young people; however, to safeguard the existence of a dating experience, it was decided to exclude those who stated that they had not been in any romantic relationship in the last 12 months.

The final sample consisted of 1,001 participants between 18 and 25 years of age, with a mean age of 21.01 years (SD = 2.093), of whom 86% (*n* = 861) were female and 13.4% (*n* = 134) were male. In relation to the sexual orientation of the participants, 82.8% identified themselves as heterosexual, followed by 11.6% Bisexual, 2.6% Pansexual and 1.8% Homosexual. In turn, 63.8% stated that they were in an exclusive relationship (single partner), while 26.3% were single without occasional partners. The occupation of most of the participants was higher education students (77.5%), 8% were workers and 8.6% engaged in both activities.

The participants of this research were invited through social networks. The invitation was disseminated publicly and specified the inclusion criteria: (1) being between 18 and 25 years old and (2) permanent residence in a region of Chile. Data was collected by convenience sampling method during the years 2021–2022.

### 2.3. Instruments

#### 2.3.1. Cyber-violence scale in adolescent couples (Cib-VPA)

Developed by Cava and Buelga (2018). This instrument is an adaptation of the scale, the original version of which is composed of 20 4-point Likert-type attitudinal/behavioral statements (1 = never; 2 = sometimes; 3 = quite often; 4 = always), intended to assess two dimensions: Cyber-harassment perpetrated, including items related to aggressive and controlling behaviors perpetrated against the partner through social networks; and Cyber-victimization, describing the same aggressive and controlling behaviors, but, in this case, assessing the extent to which adolescents have suffered such behaviors in their romantic relationship. The reliability estimates reported by Cava and Buelga (2018) are satisfactory for each dimension (ω ≥ 0.80). Additionally, the scale has been adapted and validated for use in Chilean youth (Ramírez-Carrasco et al., 2023b).

#### 2.3.2. Ambivalent sexism inventory

A 6-point, 22-item Likert-type bifactor scale designed to assess two dimensions: hostile sexism and benevolent sexism. The scale has a Cronbach’s Alpha of 0.84 and has been adapted and validated for use with university students in northern Chile (Cárdenas et al., 2010).

#### 2.3.3. Myths of romantic love scale (E-MAR)

Developed by Ramírez-Carrasco et al. (2023a). Self-report scale composed of 40 items of 4-point Likert-type behavioral/attitudinal statements (strongly disagree, disagree, agree, agree, strongly agree), intended to evaluate 8 dimensions: 1) myth of the better half (5 items); 2) myth of pairing (5 items); 3) exclusivity and fidelity myth (5 items); 4) jealousy myth (7 items); 5) omnipotence myth (4 items); 6) free will myth (4 items); 7) myth marriage (6 items); and 8) myth eternal passion (4 items). The scale presented a suitable fit [CFI = 0.969, TLI = 0.951, RMSEA = 0.047(0.045–0.050)].

#### 2.3.4. Multidimensional scale of jealousy in dating

Self-developed scale (Supplementary material), whose final version is composed of 20 items of 4-point Likert-type behavioral/attitudinal statements (1 = never; 2 = sometimes; 3 = quite often; 4 = always), designed to evaluate 4 dimensions: 1) affective jealousy related to deception (6 items); 2) affective jealousy related to abandonment (3 items); 3) cognitive jealousy (6 items); 4) behavioral jealousy (5 items). This scale has evidence of validity based on internal structure [CFI = 0.988, TLI = 0.980, RMSEA = 0.038(0.032–0.043)]. However, the behavioral jealousy dimension was excluded from the present study, given that there is a very close correspondence of the items with the dependent variable since 3 allude to online control strategies.

### 2.4. Procedure

The present study and the data collection instruments were assessed and approved by the Scientific Ethical Committee of the Universidad de Tarapacá, following the ethical Helsinki guidelines according to the World Medical Association for research with human beings.

The application of the scale was carried out in a general population of young Chileans (*N* = 1,360). The instrument was administered online together with other scales in the framework of a more extensive study, with the objective of developing an integrative-comprehensive model of dating violence in young couples and adolescents. The model included variables, such as, cyber-violence, self-esteem, jealousy, romantic love myths, sexism, emotional regulation and risk behaviors. As well as the informed consent, where the participant declared whether they accepted to participate voluntarily in the research, in which the objectives of the research, the rights of the participants, commitment to anonymity, confidentiality and use of the information for the ole purposes of the research were informed.

### 2.5. Statistical analysis

First, an exploratory structural equation analysis (ESEM) with GEOMIN rotation (Asparouhov and Muthén, 2009) was performed for the multidimensional models to assess the adequacy of the measurement models to the study sample, and the weighted least squares robust least squares estimation method (WLSMV), which has evidenced to function adequately with non-normal discrete variables and has a suitable performance for ordinal variables such as those of the instruments used to assess the constructs of interest (Suh, 2015). Confirmatory factor analyzes (CFA) and the weighted least squares robust weighted least squares estimation method (WLSMV) were performed for unidimensional models. From these analyzes, an *ad-hoc* adjustment was made to the ambivalent sexism inventory, eliminating 3 items that presented relevant cross-loadings (>0.3) or minor factorial saturations (<0.4), which produced a relevant misfit of the measurement model.

**Figure 1:**
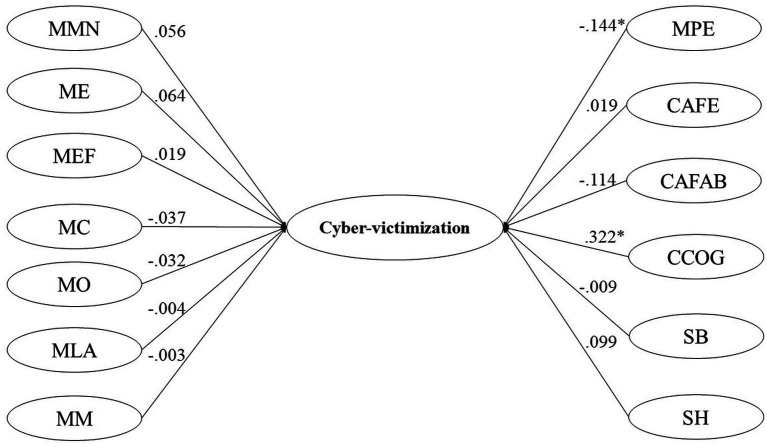
Cyber-victimization general model. MMN, myth of the better half; ME, mith of pairing; MEF, exclusivity and fidelity myth; MC, jealousy myth; MO, omnipotence myth; MLA, free will myth; MM, myth marriage; MPE, myth eternal passion; CAFE, affective jealousy related to deception; CAFAB, affective jealousy related to abandonment; CCOG, cognitive jealousy; SB, benevolent sexism; SH, hostile sexism. The coefficients are standardized.

**Figure 2:**
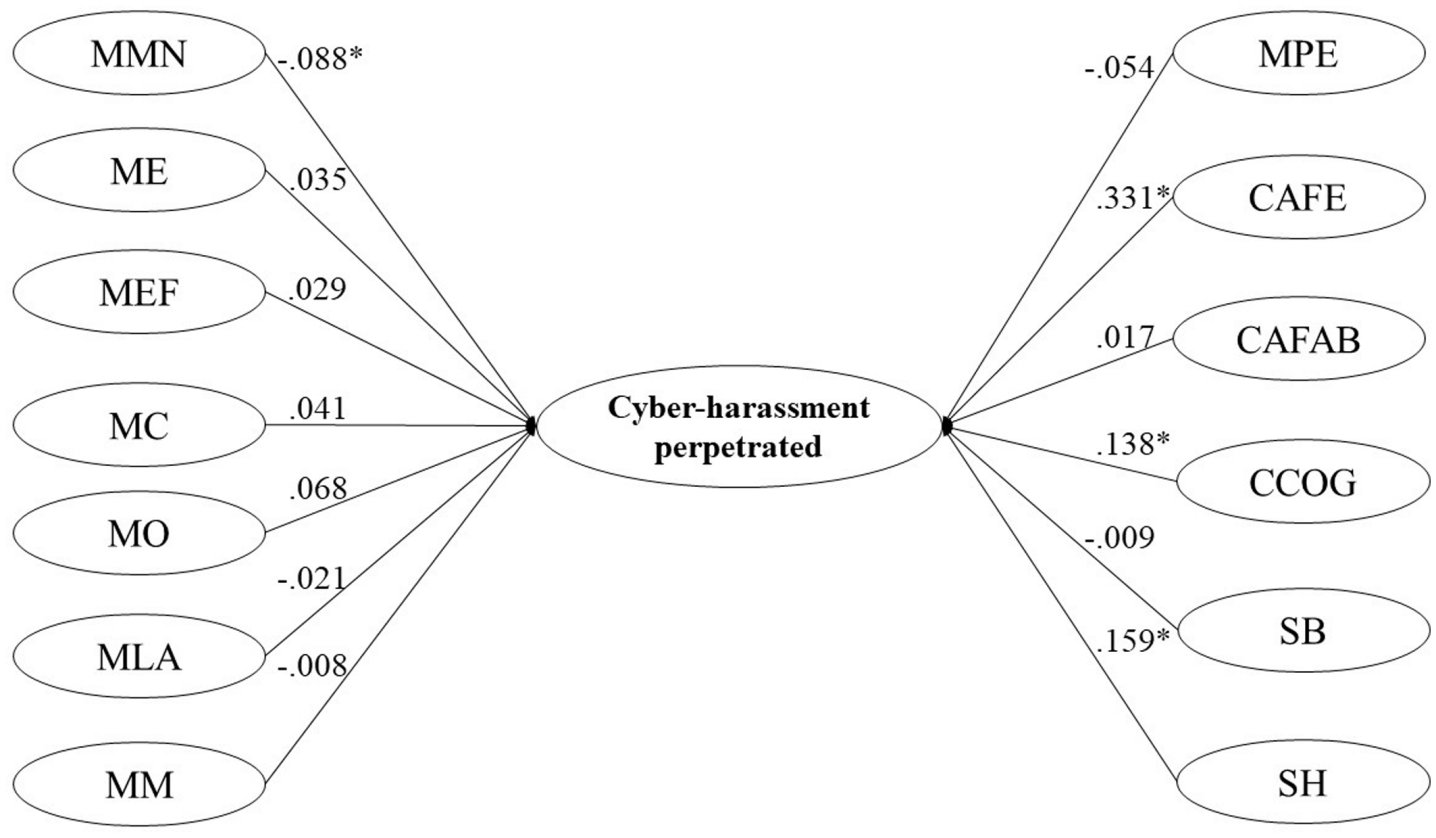
Cyber-harassment perpetrated General Model. MMN, myth of the better half; ME, mith of pairing; MEF, exclusivity and fidelity myth; MC, jealousy myth; MO, omnipotence myth; MLA, free will myth; MM, myth marriage; MPE, myth eternal passion; CAFE, affective jealousy related to deception; CAFAB, affective jealousy related to abandonment; CCOG, cognitive jealousy; SB, benevolent sexism; SH, hostile sexism. The coefficients are standardized.

Finally, using the weighted least squares robust least squares estimation method (WLSMV), 2 structural equation models were performed, one for Cyber-victimization and the other for Cyber-harassment perpetrated (see Figures 1, 2), using as independent variables the subdimensions of the measurement instruments: Ambivalent Sexism Inventory, Myths of Romantic Love Scale, and Jealousy Scale. The overall model fit was assessed following the cut-point recommendation (e.g., CFI > 0.95; TLI > 0.95; RMSEA<0.06) proposed by Schreiber (2017).

All the analyzes were carried out from the polychoric correlation matrices, which are suitable for treating ordinal variables (Barendse et al., 2015), using Mplus software version 8.2.

## 3. Results

First, global fit indicators of the measurement models for each instrument are presented (Table 1).

**Table 1:** Global fit indicators of the measurement models.

Measurement model	N° Par	χ^2^	DF	p	CFI	TLI	RMSEA	RMSEA CI 90%		SRMR
								Low	Up	
*Romantic love myths scale*										
ESEM (40 ítems; 8 dimensions)	412	1589.427	488	0.000	0.969	0.951	0.047	0.045	0.050	0.022
*Cyber-violence scale in adolescent couples*										
Cyber-harassment perpetrated										
CFA unifactorial (7 ítems)	27	31.571	14	0.0046	0.963	0.945	0.035	0.019	0.052	0.060
Cyber-victimization										
CFA unifactorial (8 ítems)	32	198.705	20	0.0000	0.971	0.960	0.094	0.083	0.107	0.065
*Ambivalent Sexism Inventory*										
ESEM (22 ítems; 2 dimensions)	109	609.736	188	0.0000	0.978	0.973	0.047	0.043	0.052	0.037
ESEM (19 ítems; 2 dimensions)	94	490.609	134	0.0000	0.977	0.970	0.052	0.047	0.056	0.034
*Multidimensional scale of jealousy in dating*										
ESEM (20 ítems; 4 dimensions)	134	280.918	116	0.0000	0.988	0.980	0.038	0.032	0.043	0.024

N Par, number of parameters in the model; *χ*^2^, jí-squared; DF, degrees of freedom; P, probability of chi-squared; CFI, comparative fit index; TLI, Tucker-Lewis index; RMSEA, mean square error of approximation; RMSEA CI 90%; SRMR, Standardized Root Mean Square Residual.

The observed values (see Table 1) mirror fit indicators adequate to the recommended standards (CFI > 0.95; TLI > 0.95; RMSEA <0.06: Schreiber, 2017), which would indicate that the measurement models are a population-based explanation of the relationships observed in this sample so that they could be incorporated into a structural equation model.

Table 2 presents the global fit indicators of the structural equation models for Cyber-victimization (see Figure 1) and Cyber-harassment perpetrated dimensions (see Figure 2). Both models showed adequate levels of fit indicators.

**Table 2:** Global fit indicators of measurement models.

Measurement model	N° Par	χ^2^	DF	p	CFI	TLI	RMSEA	RMSEA CI 90%		SRMR
								Low	Up	
*Cyber-harassment perpetrated general model*										
Covariate model	413	5340.718	3,069	0.0000	0.955	0.953	0.027	0.026	0.028	0.062
*Cyber-victimization general model*										
Covariate model	419	5328.488	3,148	0.0000	0.960	0.957	0.026	0.025	0.028	0.051

N Par, number of parameters in the model; *χ*^2^, jí-squared; DF, degrees of freedom; P, probability of chi-squared; CFI, comparative fit index; TLI, Tucker-Lewis index; RMSEA, mean square error of approximation; RMSEA CI 90%; SRMR, Standardized Root Mean Square Residual.

Table 3 shows the effects of romantic love myths, Jealousy, and sexism on cyber-violence. In the Cyber-victimization model (see Figure 1), it was observed that cognitive Jealousy had a moderate direct effect (>0.3; Cohen, 1989) on cyber-victimization, whereas the myth of eternal passion had a slight inverse effect (>0.1; Cohen, 1989).

**Table 3:** Effects of myths, jealousy and sexism on perceived and perpetrated violence.

	Cyber-victimization		Cyber-harassment perpetrated	
	Effect	*p*-value	Effect	*p*-value
Myth of the better half	0.056	0.320	−0.088*	0.028
Mith of pairing	0.064	0.286	0.035	0.370
Exclusivity and fidelity myth	0.019	0.727	0.029	0.534
Jealousy myth	−0.037	0.584	0.041	0.344
Omnipotence myth	−0.032	0.576	0.068	0.113
Free will myth	−0.004	0.934	−0.021	0.515
Myth marriage	−0.003	0.958	−0.008	0.853
Myth eternal passion	−0.144*	0.008	−0.054	0.177
Affective jealousy related to deception	0.019	0.838	0.331*	0.000
Affective jealousy related to abandonment	−0.114	0.122	0.017	0.683
Cognitive jealousy	0.322*	0.000	0.138*	0.005
Benevolent Sexism	−0.009	0.933	−0.009	0.892
Hostile sexism	0.099	0.289	0.159*	0.006

*p < 0.05.

In the Cyber-harassment perpetrated model (see Figure 2), a moderate direct effect was observed on affective Jealousy relative to cheating and mild direct effects of cognitive Jealousy and hostile sexism. Although, given the sample size, a statistically significant effect of the better half myth is observed, its practical effect is null (>, 1; Cohen, 1989).

## 4. Discussion

This research aims to identify the shared effects that beliefs associated with dating violence (romantic love myths, jealousy, and sexism) have on cyber-violence victimization and perpetration. Overall, the results showed that, when analyzed from the combined effects, some dimensions of jealousy participate in both Cyber-victimization and Cyber-harassment perpetrated. In contrast, sexist beliefs only participate in perpetration, indicating that cyber-violence is related to the probability of assault but not the probability of being a victim. In the case of romantic love myths, no relevant effects were evidenced other than a slight protective role in the cyber-victimization of the myth of eternal passion.

In the case of cyber-victimization, it was observed that cognitive jealousy seems to increase the probability of suffering virtual aggression, which could be explained by the fact that people with a tendency to this type of cognition tend to tolerate relationships prone to more abusive situations. However, considering that the study is cross-sectional and correlational, it is impossible to establish with certainty a specific directionality of the effects. Consequently, it is not possible to establish causality, and there may be bidirectional effects. In this sense, another possibility is that experiencing cyber-victimization leads to increased cognitive jealousy by generating higher uncertainty about the relationship.

In the case of Cyber-harassment perpetrated, it was observed that affective jealousy related to cheating, but not to abandonment, plays a dominant role in the manifestation of virtual aggression. One possible explanation for this is that cheating-related effects are associated with high-activation negative emotions (e.g., anger), which are strongly associated with aggression. In contrast, affective jealousy related to abandonment would be linked to low-activation negative emotions (e.g., sadness). Additionally, to affective jealousy related to cheating and cognitive jealousy, and according to the literature (Branson and March, 2021; Toplu-Demirtaş et al., 2022), hostile sexism would increase the perpetration of cyber-violence by constituting a series of attitudes that favor and legitimize violence towards women, favoring adverse effects of high activation, by making negative attributions about women’s intentions (Orozco Vargas et al., 2022).

The present study is subject to some limitations typical of this type of design. The first of these refers to the social desirability biases of the participants, which were minimized through anonymity, mainly in the dimension that alludes to the perpetration of violence against the partner. Another limitation of this study is the typical restrictions of a non-probabilistic and self-administered sampling via social networks. The sample was mainly made up of women, which could lead to a bias in the data obtained by not collecting equivalent information on men and women. An explanation for this asymmetry of the sample is that women are more willing to participate in gender-based violence issues (Bolaños, 2015; Menéndez, 2017) and have a greater inclination to report acts of GBV compared to men (Rodríguez, 2014).

Future research should consider clarifying whether the proposed model presents possible differential effects by gender, given that there are discrepancies in the results obtained in this area, with more severe consequences evidenced in the female gender (Stonard, 2021), as well as a higher probability of being identified as victims (Taquette and Monteiro, 2019). Likewise, studies in this regard highlight that dating violence is more prevalent because it is exercised in a bidirectional manner (Rojas-Solís and Romero-Méndez, 2022), an aspect that requires further study, inviting the development of comparative analyzes of both perpetration and victimization of dating violence.

Finally, the findings of this study can be a reference to guide studies on dating violence in young couples and adolescents, as well as to develop prevention and intervention programs in the educational and health care settings, given that young people and adolescents come first to consult and seek help in this context (Valdivia-Peralta et al., 2019), therefore it is essential not to make this issue invisible and to implement actions and public policies that address these variables with the aim of establishing preventive models of dating violence in order to promote healthy loving relationships in young people and adolescents.

## Data availability statement

The raw data supporting the conclusions of this article will be made available by the authors, without undue reservation.

## Ethics statement

The studies involving humans were approved by Scientific Ethical Committee of the Universidad de Tarapacá. The studies were conducted in accordance with the local legislation and institutional requirements. The participants provided their written informed consent to participate in this study. Written informed consent was obtained from the individual(s) for the publication of any potentially identifiable images or data included in this article.

## Author contributions

DR-C: contributed to conception, design of the study, performed the statistical analysis, organized the database, wrote the first draft of the manuscript, and wrote sections of the manuscript. RF-U: contributed to design of the study, performed the statistical analysis, and wrote sections of the manuscript. FP-C: contributed to write sections of the manuscript. All authors contributed to the article and approved the submitted version.

## Funding

This research was carried out with funding from the National Agency for Research and Development (ANID), a complementary benefit grant for operational expenses for a doctoral thesis research study, project code 21201938.

## Conflict of interest

The authors declare that the research was conducted in the absence of any commercial or financial relationships that could be construed as a potential conflict of interest.

## Publisher’s note

All claims expressed in this article are solely those of the authors and do not necessarily represent those of their affiliated organizations, or those of the publisher, the editors and the reviewers. Any product that may be evaluated in this article, or claim that may be made by its manufacturer, is not guaranteed or endorsed by the publisher.
