# Supplementary material for: Jealousy, sexism, and romantic love myths: the role of beliefs in online dating violence
Source: Front Psychol. 2023 Sep 4;14:1212737. doi: 10.3389/fpsyg.2023.1212737 (PMC10507330; doi:10.3389/fpsyg.2023.1212737)
Supplement: Supplementary file 1 [file Data_Sheet_1.docx]

Supplementary Material

# Supplementary Data

**Appendix A**

**Multidimensional scale of jealousy in dating (English versión)**

The following is a series of thoughts, feelings, and behaviors that some people experience in dating relationships. Please indicate how often you have, or have not, experienced each of these, indicating:

**1** never; **2** sometimes; **3** quite often; and **4** always.

|  |  | **1** | **2** | **3** | **4** |
| --- | --- | --- | --- | --- | --- |
| 1 | I worry that I might get dumped for someone else. |  |  |  |  |
| 2 | It bothers me that my partner hangs out with people who might be attractive. |  |  |  |  |
| 3 | It makes me feel bad if my partner goes to a party without me. |  |  |  |  |
| 4 | I want to know who my partner is with at all times. |  |  |  |  |
| 5 | It bothers me when my partner talks about the attractiveness of famous people. |  |  |  |  |
| 6 | I get alarmed when my partner finds other people attractive. |  |  |  |  |
| 7 | It makes me uneasy not knowing who my partner is talking to on social networks. |  |  |  |  |
| 8 | I am afraid that my partner will be interested in someone else. |  |  |  |  |
| 9 | I am afraid that my partner will leave me. |  |  |  |  |
| 10 | I think my partner is secretly seeing ex-partners. |  |  |  |  |
| 11 | I think my partner would cheat on me if I am careless. |  |  |  |  |
| 12 | I think my partner deletes compromising conversations. |  |  |  |  |
| 13 | I think my partner denies our relationship in front of other people. |  |  |  |  |
| 14 | I suspect my partner flirts with other people. |  |  |  |  |
| 15 | I suspect my partner is lying to me. |  |  |  |  |
| 16 | I check my partner's phone and social media. |  |  |  |  |
| 17 | I have asked my partner to delete or block contacts from social networks. |  |  |  |  |
| 18 | I have forbidden my partner to maintain contact with people he/she finds attractive. |  |  |  |  |
| 19 | I ask my partner to tell me what he/she does when he/she is not with me. |  |  |  |  |
| 20 | When I suspect something, I ask my partner for explanations. |  |  |  |  |

**Appendix B**

**Escala multidimensional de celos en el noviazgo (Spanish versión)**

A continuación, se presentan una serie pensamientos, sentimientos y conductas que algunas personas experimentan, cuando están en relaciones de pareja. Por favor, indica con qué frecuencia has experimentado, o no, cada una de ellas, indicando:

**1** nunca; **2** algunas veces; **3** bastantes veces; y **4** siempre.

|  |  | **1** | **2** | **3** | **4** |
| --- | --- | --- | --- | --- | --- |
| 1 | Me preocupa que me abandone por otra persona. |  |  |  |  |
| 2 | Me molesta que se junte con gente que le pudiese ser atractiva. |  |  |  |  |
| 3 | Me hace sentir mal si se va a una fiesta sin mí. |  |  |  |  |
| 4 | Quisiera saber con quién está a cada momento. |  |  |  |  |
| 5 | Me molesta cuando habla del atractivo de personas famosas. |  |  |  |  |
| 6 | Me pongo alerta cuando encuentra atractivo/a a otras personas. |  |  |  |  |
| 7 | Me pone intranquilo/a no saber con quién habla por redes sociales. |  |  |  |  |
| 8 | Me da miedo que mi pareja se interese por alguien más. |  |  |  |  |
| 9 | Me da miedo que mi pareja me abandone. |  |  |  |  |
| 10 | Creo que se ve con exparejas en secreto. |  |  |  |  |
| 11 | Creo que me engañaría si me descuido. |  |  |  |  |
| 12 | Creo que elimina conversaciones comprometedoras. |  |  |  |  |
| 13 | Creo que niega nuestra relación ante otras personas. |  |  |  |  |
| 14 | Sospecho que coquetea con otras personas. |  |  |  |  |
| 15 | Sospecho que mi pareja me miente. |  |  |  |  |
| 16 | Reviso el teléfono y/o las redes sociales de mi pareja. |  |  |  |  |
| 17 | Le he pedido que borre o bloquee contactos de redes sociales. |  |  |  |  |
| 18 | Le he prohibido que mantenga contacto con personas que le parezcan atractivas. |  |  |  |  |
| 19 | Le pido que me cuente lo que hace cuando no está conmigo. |  |  |  |  |
| 20 | Cuando sospecho algo, le pido explicaciones a mi pareja. |  |  |  |  |

**Appendix C**

**Romantic Love Myths Scale (English version)**

The following are a series of statements, with which some people agree and others disagree. Please indicate to what extent you share these ideas, indicating:

**1** if you strongly disagree; **2** if you disagree; **3** if you agree; and **4** if you strongly agree.

|  |  | **1** | **2** | **3** | **4** |
| --- | --- | --- | --- | --- | --- |
| 1 | Somewhere out there is a better half for me. |  |  |  |  |
| 2 | There is only one person made to be with me. |  |  |  |  |
| 3 | Somewhere, there is someone made to be with each person. |  |  |  |  |
| 4 | Somewhere, there is someone who completes us. |  |  |  |  |
| 5 | I am predestined to be with one person. |  |  |  |  |
| 6 | Being single is unpleasant to me. |  |  |  |  |
| 7 | If I am not in a loving relationship, I feel lonely. |  |  |  |  |
| 8 | Being single makes me feel frustrated. |  |  |  |  |
| 9 | I am afraid of not finding a partner. |  |  |  |  |
| 10 | I am afraid of being an eternal bachelor or bachelorette. |  |  |  |  |
| 11 | My partner's scenes of jealousy are signs of love. |  |  |  |  |
| 12 | If my boyfriend/girlfriend is not jealous of me, it is because he/she no longer loves me. |  |  |  |  |
| 13 | Jealousy is synonymous with true love. |  |  |  |  |
| 14 | My partner must be a jealous person to make me feel loved |  |  |  |  |
| 15 | Jealousy is a sign of love. |  |  |  |  |
| 16 | If my partner is jealous of me, it means that he/she cares a lot about me. |  |  |  |  |
| 17 | Having jealous scenes sometimes keeps the relationship alive. |  |  |  |  |
| 18 | When you love a partner, you can't like anyone else. |  |  |  |  |
| 19 | If you love someone, you don't feel like being with anyone else. |  |  |  |  |
| 20 | If my partner tells me that he/she likes someone else, I would feel that he/she does not love me. |  |  |  |  |
| 21 | Being in love implies loving only your partner. |  |  |  |  |
| 22 | You should flirt only with your partner. |  |  |  |  |
| 23 | True love can overcome any obstacle. |  |  |  |  |
| 24 | Love is enough to solve any problems. |  |  |  |  |
| 25 | Love can overcome anything. |  |  |  |  |
| 26 | Love is enough to solve all couple’s problems. |  |  |  |  |
| 27 | True love leads to marriage. |  |  |  |  |
| 28 | We must stand up for our marriage. |  |  |  |  |
| 29 | Love is the basis of marriage. |  |  |  |  |
| 30 | Love must be consolidated in marriage. |  |  |  |  |
| 31 | Marriage is the stable union of the couple. |  |  |  |  |
| 32 | Marriage is synonymous with accomplishment in love. |  |  |  |  |
| 33 | Society imposes on me whom I should like. |  |  |  |  |
| 34 | My close circle influences my decision to be with someone in a relationship. |  |  |  |  |
| 35 | Society imposes a prototype of a person I should like. |  |  |  |  |
| 36 | Family influences my choice of partner. |  |  |  |  |
| 37 | The relationship in the first few months is the same as time passes. |  |  |  |  |
| 38 | When one is in love, love remains with the same intensity throughout the relationship. |  |  |  |  |
| 39 | Love for your partner never changes. |  |  |  |  |
| 40 | The love you feel the first few months does not change over time. |  |  |  |  |

**Appendix D**

**Escala Mitos del Amor Romántico (Spanish version)**

A continuación, se presentan una serie de afirmaciones, con las que algunas personas están de acuerdo y otras no. Por favor señale en qué medida compartes estas ideas, indicando:

**1** si estás totalmente en desacuerdo; **2** si estás en desacuerdo; **3** si estás de acuerdo; y **4** si estás totalmente de acuerdo.

|  |  | **1** | **2** | **3** | **4** |
| --- | --- | --- | --- | --- | --- |
| 1 | En alguna parte existe una media naranja para mí. |  |  |  |  |
| 2 | Existe una única persona hecha para estar conmigo. |  |  |  |  |
| 3 | En alguna parte hay alguien hecho para estar con cada persona. |  |  |  |  |
| 4 | En alguna parte existe alguien que nos completa. |  |  |  |  |
| 5 | Estoy predestinado/a para estar con una persona. |  |  |  |  |
| 6 | Estar soltero/a me resulta desagradable. |  |  |  |  |
| 7 | Si no estoy en una relación amorosa me siento sola/o. |  |  |  |  |
| 8 | La soltería me hace sentir frustrado/a. |  |  |  |  |
| 9 | Tengo miedo a no encontrar pareja. |  |  |  |  |
| 10 | Tengo miedo a ser un/a eterno/a solterón/a. |  |  |  |  |
| 11 | Las escenas de celos de mi pololo/a son muestras de amor. |  |  |  |  |
| 12 | Si mi pololo/a no me cela es porque ya no me ama. |  |  |  |  |
| 13 | Los celos son sinónimos de amor verdadero. |  |  |  |  |
| 14 | Mi pareja debe celarme para sentir que me ama. |  |  |  |  |
| 15 | Los celos son un signo de amor. |  |  |  |  |
| 16 | Si mi pareja me cela, significa que le importo mucho. |  |  |  |  |
| 17 | Tener escenas de celos a veces, mantiene viva la relación. |  |  |  |  |
| 18 | Cuando se ama a una pareja no te puede gustar nadie más. |  |  |  |  |
| 19 | Si amas a alguien, no sientes deseos de estar con nadie más. |  |  |  |  |
| 20 | Si mi pareja me dice que le gusta otra persona, sentiría que no me ama. |  |  |  |  |
| 21 | Estar enamorado implica amar solamente a tu pareja. |  |  |  |  |
| 22 | Se debe coquetear solo con la propia pareja. |  |  |  |  |
| 23 | El amor verdadero puede superar cualquier obstáculo. |  |  |  |  |
| 24 | Es suficiente el amor para solucionar los problemas. |  |  |  |  |
| 25 | El amor puede vencer cualquier cosa. |  |  |  |  |
| 26 | El amor es suficiente para solucionar todos los problemas que tenga una pareja. |  |  |  |  |
| 27 | El amor verdadero conduce al matrimonio. |  |  |  |  |
| 28 | Hay que luchar por el matrimonio. |  |  |  |  |
| 29 | El amor es la base del matrimonio. |  |  |  |  |
| 30 | El amor debe consolidarse en el matrimonio. |  |  |  |  |
| 31 | El matrimonio es la unión estable de la pareja. |  |  |  |  |
| 32 | El matrimonio es sinónimo de triunfo en el amor. |  |  |  |  |
| 33 | La sociedad me impone quien me debe gustar. |  |  |  |  |
| 34 | Mi círculo cercano influye en mi decisión de estar con alguien en una relación. |  |  |  |  |
| 35 | La sociedad impone un prototipo de persona que me debería gustar. |  |  |  |  |
| 36 | La familia influye en la elección de mi pareja. |  |  |  |  |
| 37 | La relación de los primeros meses es igual a medida que pasa el tiempo. |  |  |  |  |
| 38 | Cuando se está enamorado, el amor se mantiene con la misma intensidad durante toda la relación. |  |  |  |  |
| 39 | El amor por la pareja nunca cambia. |  |  |  |  |
| 40 | El amor de los primeros meses no cambia con el tiempo. |  |  |  |  |
